# Functional Characterization of Trypsin Genes in *Spodoptera litura* and Their RNA Silencing in Combination with Insecticides

**DOI:** 10.3390/insects17080861

**Published:** 2026-08-18

**Authors:** Li Xu, Ke Wang, Yin Huang, Yunsheng Wei, Hao Yu, Runqiang Liu, Dongzhi Li

**Affiliations:** 1 College of Plant Protection and Environment, Henan Institute of Science and Technology, Xinxiang 453003, China; 2 Henan Engineering Research Center of Green Pesticide Creation & Intelligent Pesticide Residue Sensor Detection, Henan Institute of Science and Technology, Xinxiang 453003, China; 3 State Key Laboratory of High-Efficiency Production of Wheat-Maize Double Cropping, Xinxiang 453003, China

**Keywords:** *Spodoptera litura*, trypsin, RNAi, star polycation, RNA biopesticide

## Abstract

Trypsin is one of the major digestive proteinases secreted by the insect midgut. It plays important roles in digestion, molting, diapause, zymogen activation, innate immunity, reproduction and insecticide resistance. However, the functions of trypsin and its potential as a target for the control of *Spodoptera litura*, a polyphagous agricultural pest, remain poorly understood. In this study, seven trypsin genes related to *S. litura* development were characterized. Temporal-spatial expression results showed that the seven trypsin genes were predominantly expressed during the larval stage and mainly localized in the gut. Their expression was positively correlated with juvenile hormone titer. Oral RNAi using star polycation (SPc) as a nanocarrier showed that silencing *Sltrypsin1*, *Sltrypsin5*, and *Sltrypsin7* significantly reduced larval weight and food conversion efficiency and prolonged larval development. In a field-simulating trial, when *dsSltrypsin1*/SPc or *dsSltrypsin7*/SPc was sprayed on soybean or maize leaves, it significantly delayed *S. litura* larval development, decreasing their pupation and eclosion rates by 37.1–42.3% and 12.7–30.4%, respectively. The overall control efficacy ranged from 48.1% to 57.4%, suggesting that *Sltrypsin1* and *Sltrypsin7* play important roles in the digestion of soybean and maize. Feeding on artificial diets containing *dsSltrypsin1*/SPc or *dsSltrypsin7*/SPc significantly reduced LC_50_ values of lufenuron, spinosad, RH-5849, and chlorantraniliprole, with synergism ratios exceeding 1.5-fold. These results suggested that Sltrypsin1 and Sltrypsin7 play important roles in food digestion in *S. litura* feeding on soybean and maize. They could be co-applied with insecticides to increase control efficiency, reduce the application amount and mitigate resistance to conventional insecticides.

## Simple Summary

*Spodoptera litura* is a destructive pest that feeds on many crops, including soybean and maize, causing serious yield losses. In recent years, it has developed serious resistance to multiple conventional and novel insecticides. Knocking down the genes related to insecticide resistance or insect growth is a useful way to increase insecticide susceptibility. Trypsin genes play important roles in digestion, molting, diapause, zymogen activation, innate immunity, reproduction and insecticide resistance. In this study, we found that silencing the expression of two specific trypsins slowed larval growth, reduced their ability to convert food into body mass, and lowered the rates of normal pupation and adult emergence. In field-like tests on soybean and maize leaves, this approach gave over 48% pest control. The combined application of RNA silencing of these two trypsin genes and insecticide treatment significantly increased the susceptibility of *S. litura* to the insecticides, thereby reducing the amount of insecticide required. Our findings suggested that targeting these two trypsins offers a promising, environmentally friendly strategy to manage this pest, reduce crop damage, and decrease reliance on chemical sprays, which could benefit farmers and sustainable agriculture.

## 1. Introduction

RNA interference (RNAi)-based biopesticides have been hailed as the third revolution in pesticide development. They could serve as an alternative to chemical pest control techniques, decreasing the application of chemical insecticides. RNA biopesticides are primarily delivered through three approaches: spray-induced gene silencing (SIGS), host-induced gene silencing (HIGS), and virus- or bacteria-mediated gene silencing. Compared with HIGS, SIGS is more widely accepted because it is easier to apply and does not involve transgenic technology [1,2]. In 2023, the world’s first SIGS RNA biopesticide, Calantha™, was approved by the U.S. EPA, and it began commercial sales in 2024. In 2025, the U.S. EPA announced its intent to approve the second RNA biopesticide, Vadescana, developed by GreenLight Biosciences, for controlling Varroa destructor mites in honeybee colonies [2,3].

Screening high-efficiency RNAi target genes is crucial, but it is a labor-intensive and time-consuming process [4]. Genes involved in growth, development, essential cellular processes, and insecticide resistance have frequently been selected and evaluated as potential targets for RNAi-based biopesticides [3,5]. Naked dsRNA is inherently unstable, and nanocarrier-based delivery systems have therefore been developed to improve its stability and delivery efficiency. However, the application of dsRNA alone under field conditions often offers limited pest control efficacy [1,3]. The co-application of nano-formulated dsRNA with conventional insecticides may produce synergistic effects, enhancing control efficacy while reducing chemical insecticide needs. This strategy could substantially facilitate the practical adoption of RNAi-based biopesticides in field pest management [2,4,6].

Serine proteases are important digestive enzymes characterized by a serine residue in their catalytic active site. Among them, trypsin is one of the predominant digestive proteases, which plays a vital role in protein digestion in lepidopteran insects [7]. Trypsin possesses a highly conserved His-Asp-Ser catalytic triad. It cleaves peptide chains at the carboxyl groups of arginine or lysine residues, facilitating peptide bond hydrolysis. Trypsin not only provides amino acids for various physiological processes through protein digestion but also participates in molting, diapause, reproduction, zymogen activation, and innate immunity [8,9]. In *Nilaparvata lugens*, *NlTrypsin-8*, *NlTrypsin-29* and *NlTrypsin-32* were shown to be essential for ecdysis. Silencing these genes could disrupt nymphal molting by preventing the successful shedding of the exocuticle [10]. The trypsin p37k protein, purified from the molting fluid of *Bombyx mori*, could hydrolyze CPAP3-type cuticular proteins and thereby played an important role in the molting process [11]. In *Plutella xylostella*, *PxTrypsin1* was identified as a seminal fluid protein in adult males, and its deletion using CRISPR/Cas9 resulted in abnormal sperm development [12]. These findings further indicate that trypsins play important roles in insect growth, development and reproduction.

Star polycation (SPc) is a dendritic macromolecular polymer with a hydrophobic core and a hydrophilic shell, developed by researchers at China Agricultural University. It has a simple structure and low production cost, and can form stable complexes with dsRNA through noncovalent interactions [13]. Previous studies have demonstrated that SPc is an efficient nanocarrier of dsRNA, enhancing its stability and delivery efficiency, protecting it from degradation, and prolonging the duration of RNAi-mediated protection [14,15,16,17].

*Spodoptera litura* is a highly polyphagous agricultural pest that feeds on more than 100 crop and vegetable species across tropical and subtropical regions worldwide. It causes substantial economic losses, and it can cause a complete failure in severely infested cash crops [18]. Due to the extensive use of chemical insecticides, *S. litura* has developed substantial resistance to multiple conventional and newly developed insecticides [19,20,21,22]. Developing novel RNAi-based biopesticide strategies represents a promising approach for resistance management and integrated control of *S. litura*.

In our previous study, a 1,2,4-triazole derivative, ribavirin, was reported to exhibit insect growth regulator activity in *S. litura* larvae, inhibiting the expression of trypsin genes, reducing juvenile hormone (JH) titer, and impairing larval molting [23]. These trypsin genes may play important roles in larval growth and development. To clarify their biological functions and evaluate their potential as targets for *S. litura* control, RNA interference was used to assess the effects of gene silencing on larval development. The efficacy of their combined application with conventional insecticides through spray-induced gene silencing was also investigated. This study provides useful information for the development of trypsin-targeting RNA biopesticides and offers a new strategy for the environmentally sustainable management of *S. litura*.

## 2. Materials and Methods

### 2.1. Insect

*S. litura* population was kindly provided by Guangxi Tianyuan Biochemistry Crop., Ltd. (Nanning, China), and reared in an insectary with artificial diets at 27 ± 1 °C with 70 ± 10% relative humidity and a photoperiod of 14:10 (L:D) h without insecticide exposure. *S. litura* adults were supplemented with 10% honey water.

### 2.2. Chemical Reagents

Methoprene (95%) and geraniol (97%) were purchased from J&K (Beijing, China). Technical grade chlorpyrifos (95%), RH-5849 (90%), lufenuron (97%), chlorantraniliprole (95%), and emamectin benzoate (72%) were provided by Henan Jintiandi Co., Ltd. (Kaifeng, China). Technical grade spinosad (92%) and indoxacarb (98%) were provided by Henan Hansi Co., Ltd. (Shangqiu, China); 6.5% SPc nanocarrier was purchased from Beijing Qingyuanbao Biotechnology Co., Ltd. (Beijing, China); 8000 IU/μL suspension concentrate of *Bacillus thuringiensis* was purchased from Shandong Lukang Biological Pesticides Co., Ltd. (Dezhou, China).

### 2.3. Phylogenetic Analysis and Heatmap Analysis of Seven Trypsin Genes

The chromosomal locations of the trypsin genes were visualized using MG2C (http://mg2c.iask.in/mg2c_v2.1/ (accessed on 20 September 2025)). A heatmap showing the expression patterns of trypsin genes suppressed by ribavirin was generated using ChiPlot (https://www.chiplot.online/ (accessed on 16 May 2024)). Phylogenetic relationships among the trypsin genes were analyzed in MEGA 11.0 using the maximum-likelihood method with 1000 bootstrap replicates.

### 2.4. The Temporal-Spatial Expression and Induction Expression of Trypsin Genes

For temporal-spatial expression, samples including larvae of the 1st–6th instar, pupae, and cuticle, head, foregut, midgut, hindgut, Malpighian Tubules, and fat body dissected from the 5th instar larvae were collected and used for RNA extraction.

For the induction of expression, artificial diets containing methoprene or geraniol at 10 mg/kg, 50 mg/kg, and 100 mg/kg were used to feed the 3rd-instar larvae. Briefly, the stock solutions of methoprene or geraniol at 10,000 mg/L dissolved in acetone and ethanol, respectively, were prepared. A series of volumes of the stock solution were added to the artificial diets before solidification to achieve final concentrations of 10 mg/kg, 50 mg/kg, and 100 mg/kg, respectively. After being fed for 12 h, six larvae were collected for RNA extraction. Each treatment was repeated 3 times.

cDNA was synthesized with the FastKing cDNA First Strand Synthesis Kit (Tiangen, China) and used for qRT-PCR using a QuantStudio 6 Flex Real-Time PCR System. Primer pairs used for qRT-PCR are listed in Table S1. EF1α and RPL10 served as internal reference genes. Each reaction was performed in triplicate with 3 cDNA samples.

### 2.5. Synthesis of Trypsin dsRNA/SPc

The cDNA was used to amplify a 400 bp fragment of the trypsin gene (Primer pairs were listed in Table S2). PCR products were ligated into the L4440 plasmid vector (Shanghai Bolang Biotechnology Co., Ltd. (Shanghai, China) between the restriction enzyme sites Sac *I* and Apa *I*. The Trypsin-L4440 expression vector was introduced into the thawed HT115 competent *E. coli* cells by heat shock. The diluted cells were plated on LB solid medium plates containing 50 mg/mL ampicillin and 12.5 mg/mL tetracycline and incubated overnight. Single colonies were picked and inoculated into LB liquid medium. Positive clones were screened and verified by colony PCR and sequencing. The above HT115 culture carrying Trypsin-L4440 was inoculated at a 1:100 ratio into 800 mL fresh LB medium and cultured at 37 °C, 160 rpm until OD600 reached 0.6. IPTG at 1 mM was added to induce expression for 4–6 h. After that, the culture was centrifuged at 6000 rpm, 4 °C for 5 min to collect the bacterial cells. The pellet was washed once with pure water and dsRNA was extracted and purified using the MagBeads E. Coli Expressed dsRNA Extraction and Purification Kit (Shanghai Bolang Biotechnology Co., Ltd.).

The dsRNA/SPc mixture was prepared according to Chao et al. [14]. Briefly, the dsRNA was mixed with SPc at a mass ratio of 1:1 and incubated for 15 min at room temperature. The dsRNA was then adsorbed on the SPc surface by electrostatic interaction.

### 2.6. Evaluating the RNAi Effect of the Trypsin dsRNA by Feeding

The artificial diet was prepared according to Tang et al. [24] with maize flour, bean flour, yeast, ascorbic acid, sorbic acid, citric acid, casein, agar, and roxithromycin. RNAi was performed by feeding the 3rd-instar larvae with a 0.5 cm cubed artificial diet, onto which the dsRNA/SPc complex (2 μg) was loaded via surface coating. Artificial diets supplemented with pure water or dsGFP/SPc were used as controls. After being fed for 24 h and 48 h, larvae were sampled for RNA extraction. qRT-PCR was used to determine the silencing efficiency of trypsin genes. A total of 12 larvae were included in each treatment, which was repeated 3 times.

To ensure consistent RNAi efficiency, fresh diet supplemented with dsRNA/SPc (2 μg) solution was replaced every 24 h, and the weight of larvae and artificial diet was measured daily until pupation. A total of 12 larvae were included in each treatment, and the experiment was repeated 10 times. To minimize disturbance to larval development, four of the ten replicates were used to monitor larval and artificial diet weights, while the remaining six replicates were used to determine the pupation rate, duration of the pupal stage, pupal weight, and malformation rate. The amount of food consumed by each larva was determined and the food conversion efficiency was calculated according to the formula [25]:

Food Conversion Efficiency = A × 100%/B (1)

A is the weight increase in the larvae, and B is the feeding amount.

### 2.7. Field Simulation Trial and Its Influence on Host Feeding

To further evaluate its potential for field application and its role in digesting host leaves, RNAi was performed by feeding the 3rd-instar larvae with host leaves (soybean and maize) sprayed with dsRNA/SPc solution (675 L/hm2, 20 μg/mL) until pupation. Leaves sprayed with dsGFP/SPc were used as a control. Fresh leaves sprayed with dsRNA/SPc or dsGFP/SPc were replaced daily. Larval weight, pupation rate, pupae weight, malformation rate, eclosion rate and food consumed were documented. The replications were set as described in Section 2.6.

### 2.8. Effects of Silencing Sltrypsin1 or Sltrypsin7 on the Susceptibility of S. litura to Insecticides

Toxic artificial diets containing a series of insecticide concentrations were prepared. The 3rd-instar *S. litura* larvae were fed with a toxic artificial diet supplemented with dsRNA/SPc (2 μg) as described in 2.6. Larvae fed with an artificial diet supplemented with dsGFP/SPc were used as a control. Mortality was checked after 48 h of treatment. Each treatment consisted of 12 insects and was repeated 3 times.

### 2.9. Statistical Analysis

The relative expression level of trypsin genes determined by qRT-PCR was calculated using 2^−ΔΔCt^ with Ct values. Statistical differences in the temporal-spatial expression and induction expression of trypsin genes, and in *S. litura* development parameters affected by RNAi, were analyzed by one-way analysis of variation (ANOVA) followed by Tukey’s HSD test. Statistical differences in the RNAi efficiency of trypsin genes were analyzed using Student’s *t*-test. The LC_50_ values of insecticides against *S. litura* were calculated using SPSS 16.0 (SPSS, Chicago, IL, USA) with probit analysis and non-overlapping of the 95% confidence limits of LC_50_ was considered a significant difference.

## 3. Results

### 3.1. The Bioinformatics of Seven Trypsin Genes

**Figure 1:**
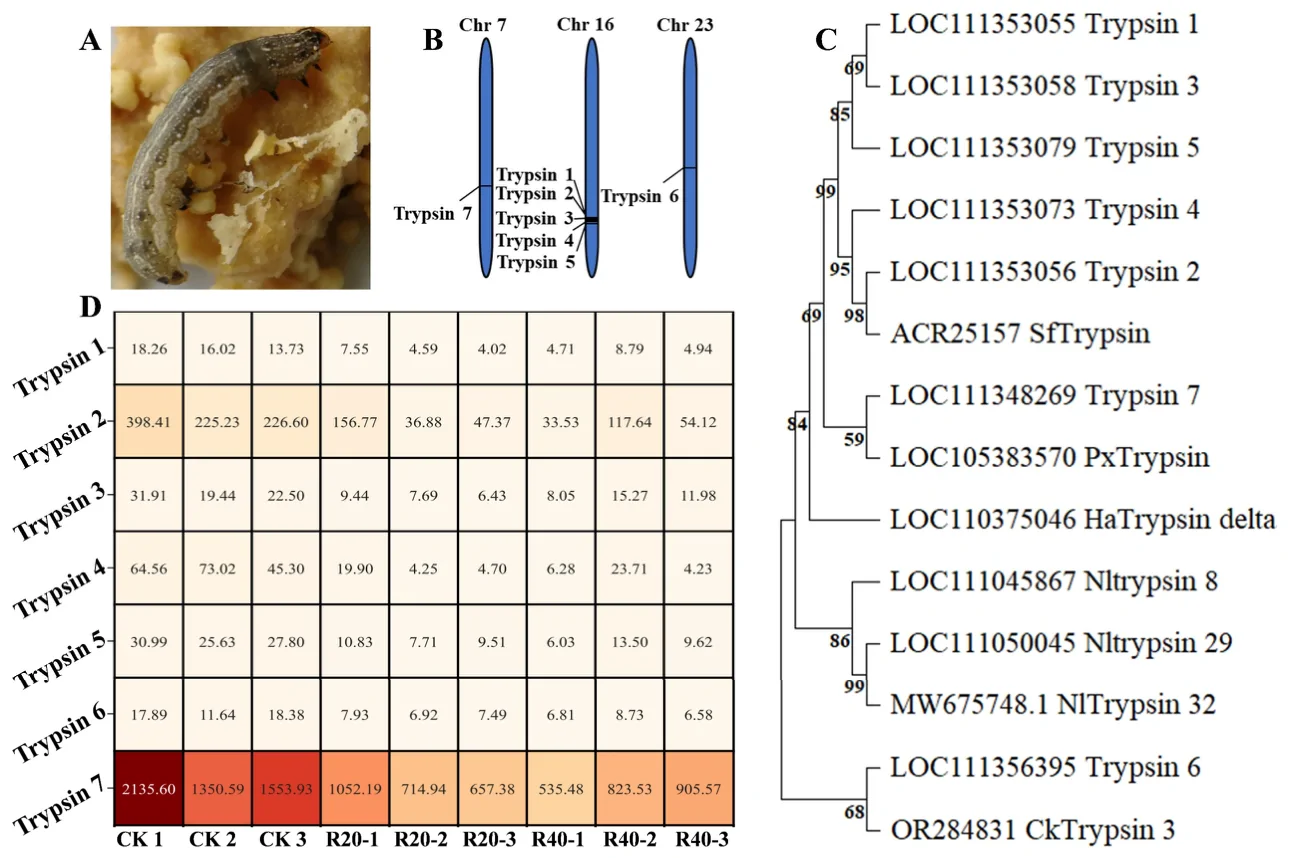
The reduced feeding in *S. litura* larvae treated with ribavirin (**A**), location on the chromosome (**B**), the phylogenetic tree (**C**), and heatmap (**D**) of 7 trypsin genes. CK indicates the control. R20 and R40 indicate ribavirin treatment at 20 mg/kg and 40 mg/kg, respectively. *Spodoptera frugiperda* (Sf), *Plutella xylostella* (Px), *Helicoverpa armigera* (Ha), *Nilaparvata lugens* (Nl), and *Chironomus kiiensis* (Ck). In (**D**), the darker the color, the higher the gene expression level.

The seven trypsin genes were selected from the transcriptome in our previous study [23]. Larvae fed on artificial diets containing ribavirin experienced reduced feeding and inhibited growth (Figure 1A). Sltrypsin1, Sltrypsin2, Sltrypsin3, Sltrypsin4, and Sltrypsin5 are located on chromosome 16. Sltrypsin6 and Sltrypsin7 are located on chromosomes 23 and 7, respectively (Figure 1B). Sltrypsin1, Sltrypsin2, Sltrypsin3, Sltrypsin4, and Sltrypsin5 clustered into one branch, indicating a close genetic relationship. Sltrypsin6 was distantly related to the other genes, suggesting a more distant genetic relationship. Sltrypsin2, Sltrypsin6 and Sltrypsin7 clustered with Sftrypsin (ACR25157), CkTrypsin3 (OR284831) and PxTrypsin (LOC105383570), respectively (Figure 1C). Their expression in the 3rd-instar larvae was inhibited by ribavirin. The abundance of Sltrypsin7 was the highest (Figure 1D).

### 3.2. The Temporal-Spatial Expression Levels of Seven Trypsin Genes

**Figure 2:**
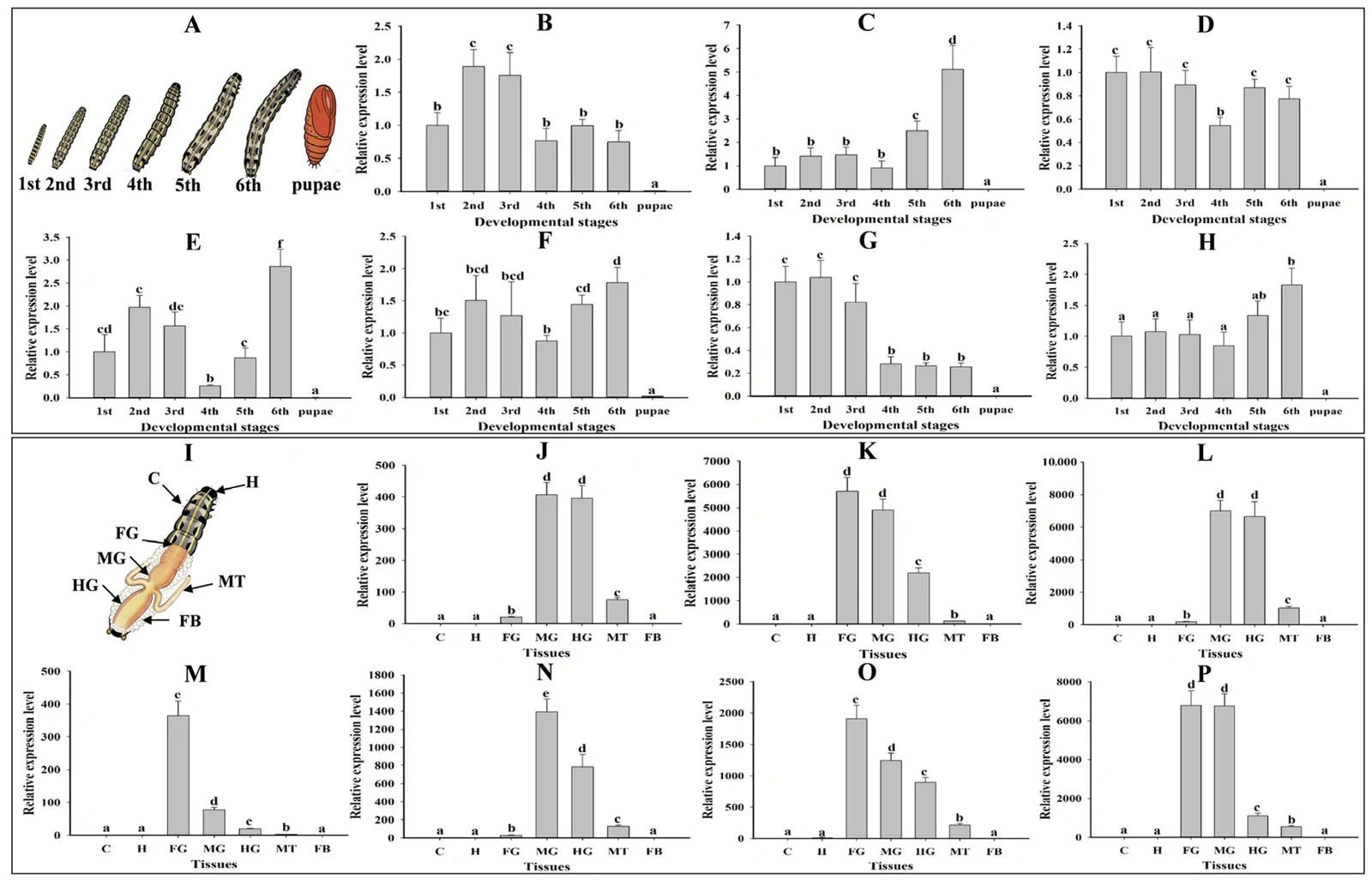
The graphical representation of samples used for temporal-spatial expression determined by qRT-PCR and the relative expression level of trypsin genes. (**A**) Graphical representation of different developmental stages of *S. litura* used for temporal expression determination. (**B**–**H**) The relative expression levels of Sltrypsin1, Sltrypsin2, Sltrypsin3, Sltrypsin4, Sltrypsin5, Sltrypsin6, and Sltrypsin7 in different developmental stages of *S. litura*. (**I**) Graphical representation of different tissues of *S. litura* larvae used for spatial expression determination. (**J**–**P**) The relative expression levels of Sltrypsin1, Sltrypsin2, Sltrypsin3, Sltrypsin4, Sltrypsin5, Sltrypsin6, and Sltrypsin7 in different tissues of 5th instar larvae of *S. litura*. Different lowercase letters above the bars indicate significant differences analyzed by ANOVA followed by Tukey’s HSD test (*p* < 0.05). Head (H), cuticle (C), foregut (FG), midgut (MG), hindgut (HG), Malpighian tubules (MT), and fat body (FB).

The expression levels of seven trypsin genes were determined across larval and pupal stages, as well as in specific tissues of 5th-instar larvae. As shown in Figure 2, all seven genes were actively expressed in larvae but were either absent or expressed at trace levels in pupae. Tissue-specific analysis revealed high expression in the gut, low expression in the Malpighian tubules, and negligible expression in the cuticle, head, and fat body. *Sltrypsin1*, *Sltrypsin3*, and *Sltrypsin5* exhibited peak expression in the midgut and hindgut, whereas *Sltrypsin2*, *Sltrypsin4*, and *Sltrypsin7* were predominantly expressed in the foregut and midgut. Additionally, the expression levels of *Sltrypsin1*, *Sltrypsin3*, *Sltrypsin5*, and *Sltrypsin7* remained relatively stable throughout the larval stage.

### 3.3. The Induction of Trypsin Genes by Methoprene and Geraniol

**Figure 3:**
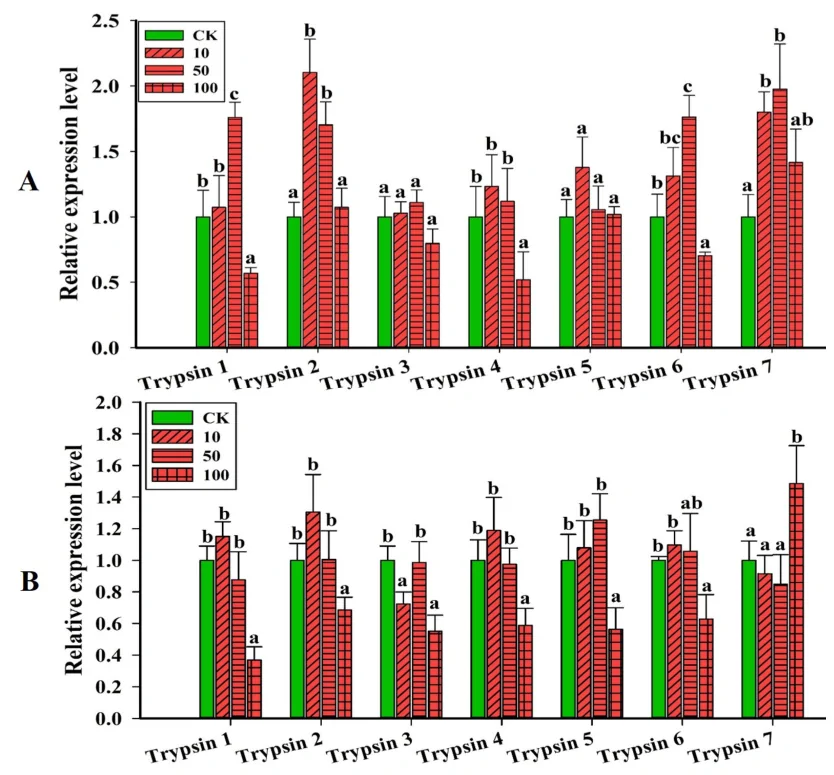
The relative expression levels of 7 trypsin genes in *S. litura* induced by methoprene (**A**) and geraniol (**B**). Different lowercase letters above the bars indicate significant differences analyzed by ANOVA followed by Tukey’s HSD test (*p* < 0.05). CK, 10, 50, and 100 indicate methoprene and geraniol concentrations of 0 mg/kg, 10 mg/kg, 50 mg/kg and 100 mg/kg, respectively.

To validate the regulation of trypsin genes by JH, the JH analog methoprene and inhibitor geraniol were used to feed larvae by mixing with artificial diets. Methoprene at 10 and 50 mg/kg significantly induced the expression of *Sltrypsin2* and *Sltrypsin7*, while 50 mg/kg methoprene also significantly upregulated *Sltrypsin1* and *Sltrypsin6*. In contrast, methoprene at 100 mg/kg significantly inhibited the relative expression of *Sltrypsin1*, *Sltrypsin4*, and *Sltrypsin6*. The expression of *Sltrypsin3* and *Sltrypsin5* was not influenced by methoprene at 10, 50 or 100 mg/kg. Geraniol at 100 mg/kg significantly inhibited the expression of *Sltrypsin1*–*Sltrypsin6*, while significantly inducing the expression of *Sltrypsin7*. The expression of all seven trypsin genes was almost not influenced by geraniol at 10 or 50 mg/kg (Figure 3).

### 3.4. The Gene Silencing Efficiency of Seven Trypsin Genes

**Figure 4:**
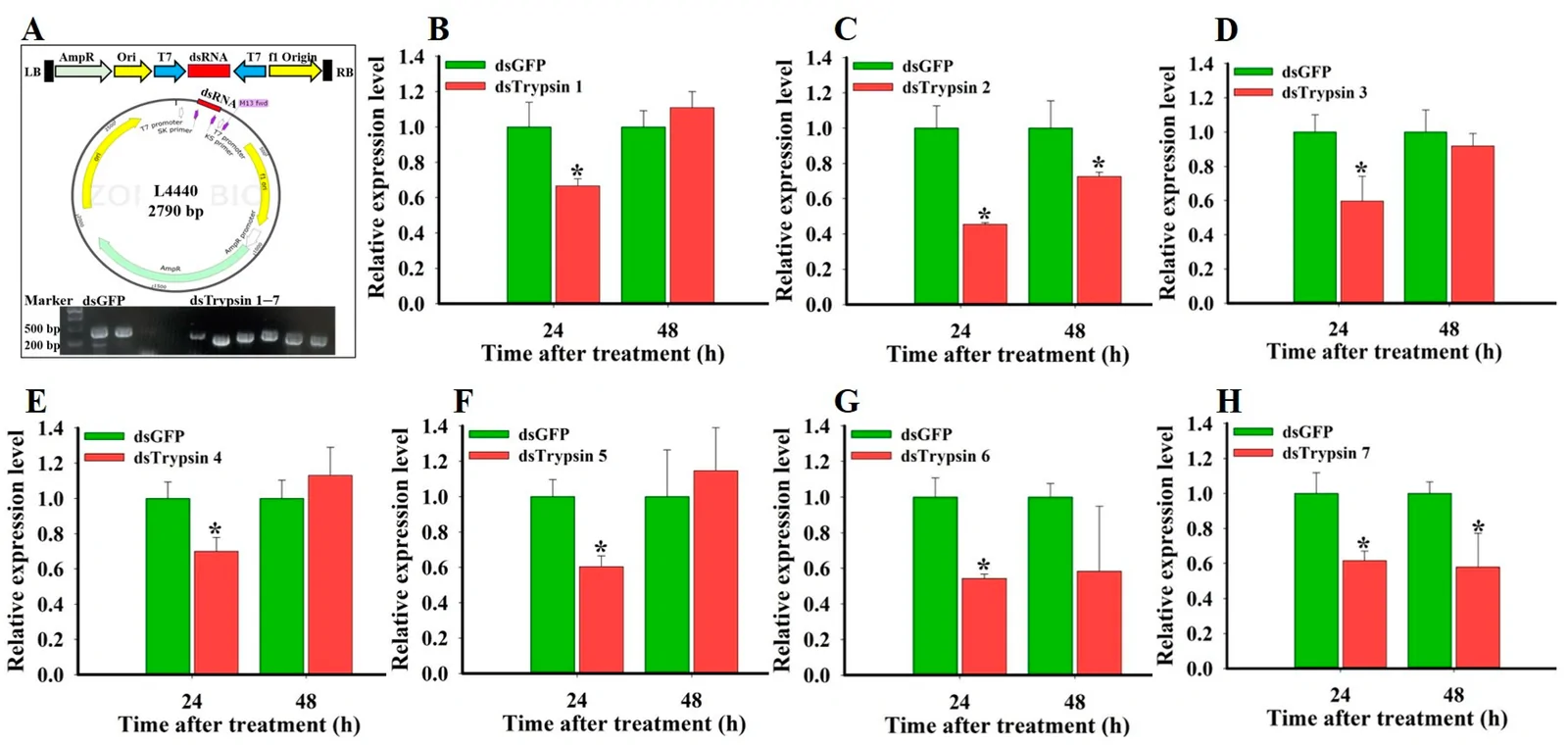
The graphical representation of the plasmid vector expressing trypsin dsRNA (**A**) and the RNAi efficiency of Sltrypsin1 (**B**), Sltrypsin2 (**C**), Sltrypsin3 (**D**), Sltrypsin4 (**E**), Sltrypsin5 (**F**), Sltrypsin6 (**G**), and Sltrypsin7 (**H**). The asterisk indicates a significant difference analyzed by Student’s *t*-test (*p* < 0.05).

The trypsin-L4440 expression vector was constructed to express the dsRNA of trypsin genes (Figure 4A). After 24 h of feeding on artificial diets containing dsSltrypsin/SPc, the expression levels of all seven trypsin genes were significantly reduced by 33.4–54.6% relative to the control (Figure 4B–H). After 48 h, the expression levels of *Sltrypsin2* and *Sltrypsin7* remained significantly lower than those in the control, i.e., by 28.3% and 42.1%, respectively, whereas the expression levels of the other genes did not differ significantly. from those in the control.

### 3.5. Effects of Trypsin Gene Silencing on the Developmental Parameters of S. litura

**Figure 5:**
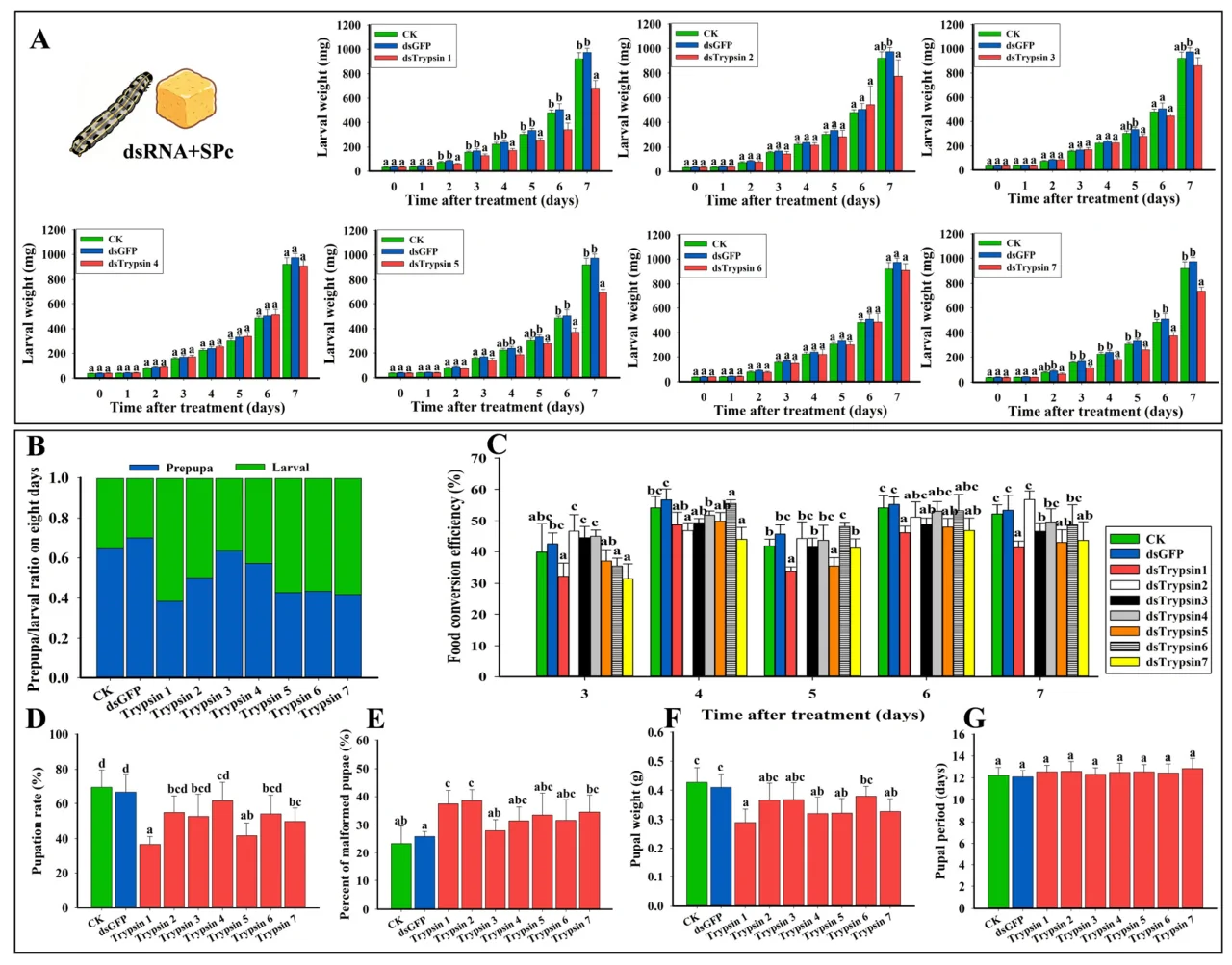
The influence on the development of *S. litura* by feeding artificial diets supplemented with dsRNA/SPc. (**A**) The monitored larval weight after feeding on artificial diets supplemented with dsRNA/SPc for 7 days. (**B**) The prepupa/larval ratio of *S. litura* after feeding on artificial diets supplemented with dsRNA/SPc for 8 days. (**C**) The food conversion efficiency of 7 trypsin genes after feeding on artificial diets supplemented with dsRNA/SPc for 3, 4, 5, 6, and 7 days. The pupation rate of *S. litura* larvae (**D**), the percentage of malformed pupae (**E**), the pupal weight (**F**) and the pupal period (**G**) after feeding on artificial diets supplemented with dsRNA/SPc. Different lowercase letters above the bars indicate significant differences analyzed by ANOVA followed by Tukey’s HSD test (*p* < 0.05).

The developmental parameters of *S. litura* were monitored continuously after being fed artificial diets containing dsRNA/SPc. Compared with larvae fed either an untreated artificial diet or a diet containing dsGFP/SPc, larval weight was significantly reduced from day 2 onward in the dsSltrypsin1/SPc treatment, from day 6 onward in the dsSltrypsin5/SPc treatment, and from day 3 onward in the dsSltrypsin7/SPc treatment. No significant changes in larval weight were observed after being fed artificial diets containing the dsRNA/SPc of the other trypsin genes (Figure 5A). After 8 days of feeding on dsRNA/SPc-containing diets, the ratio of prepupae to larvae was markedly lower than that in the untreated control and dsGFP/SPc groups, particularly following the silencing of *Sltrypsin1*, *Sltrypsin5*, *Sltrypsin6*, and *Sltrypsin7* (Figure 5B). The food conversion efficiency of larvae fed with artificial diets containing dsSltrypsin1/SPc, dsSltrypsin5/SPc and dsSltrypsin7/SPc was significantly lower than that of the control or dsGFP/SPc groups after treatment for 5, 6 and 7 days (Figure 5C). After pupation, the pupation rate and pupal weight in pupae treated with dsSltrypsin1/SPc, dsSltrypsin5/SPc and dsSltrypsin7/SPc decreased significantly compared with the control or dsGFP/SPc groups, while the malformed pupae ratio treated with dsSltrypsin1/SPc and dsSltrypsin2/SPc significantly increased (Figure 5D–F). Feeding with artificial diets containing trypsin dsRNA/SPc showed no significant influence on the pupal period (Figure 5G).

### 3.6. Digestive Function Validation in Maize and Soybean and Evaluation of Field Application Potential

**Figure 6:**
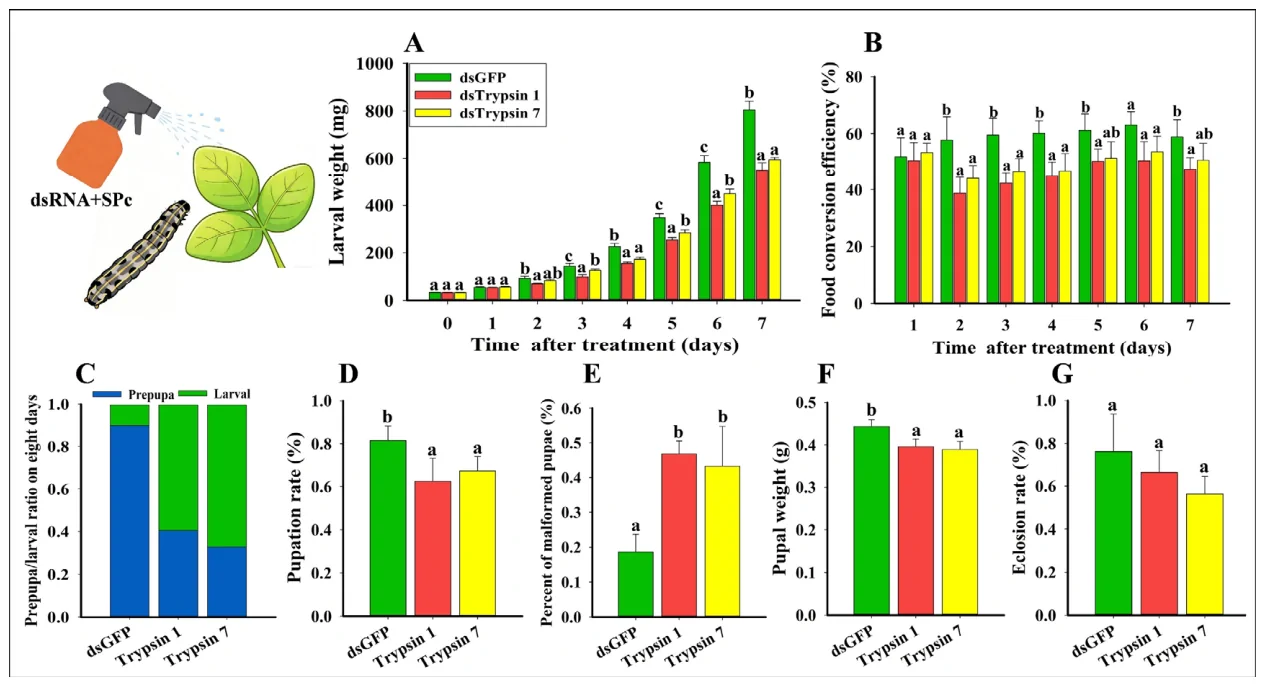
The influence on the development of *S. litura* after spraying dsSltrypsin1/SPc or dsSltrypsin7/SPc on soybean leaves. (**A**) The monitored larval weight after spraying dsSltrypsin1/SPc or dsSltrypsin7/SPc for 7 d. (**B**) The food conversion efficiency after spraying dsSltrypsin1/SPc or dsSltrypsin7/SPc for 1, 2, 3, 4, 5, 6, and 7 d. (**C**) The prepupa/larval ratio of *S. litura* after spraying dsSltrypsin1/SPc or dsSltrypsin7/SPc for 8 d. The pupation rate of *S. litura* larvae (**D**), the percentage of malformed pupae (**E**), pupal weight (**F**) and eclosion rate (**G**) after spraying dsSltrypsin1/SPc or dsSltrypsin7/SPc. Different lowercase letters above the bars indicate significant differences analyzed by ANOVA followed by Tukey’s HSD test (*p* < 0.05).

Based on the influence on *S. litura* growth and development after the silencing of trypsin genes, *Sltrypsin1* and *Sltrypsin7* were selected as key candidate genes. To further investigate the effects of silencing these genes on the growth and development of *S. litura* under field-simulated conditions involving different host plants, their roles in the digestion of soybean and maize were characterized, and their potential for field application was evaluated. The development parameters of *S. litura* were monitored continuously after being fed soybean or maize leaves sprayed with dsRNA/SPc. Compared with larvae fed with soybean leaves sprayed with dsGFP/SPc, the weight of larvae treated with ds*Sltrypsin1*/SPc and ds*Sltrypsin7*/SPc decreased significantly compared to the 2nd day (Figure 6A). The food conversion efficiency of soybean leaves treated with ds*Sltrypsin1*/SPc was significantly lower than that of dsGFP/SPc after treatment for 2, 3, 4, 5, and 7 days, while that of ds*Sltrypsin7*/SPc was significantly lower only after treatment for 2, 3, and 4 days (Figure 6B). After 8 days of treatment, the prepupa-to-larva ratio was markedly lower in the ds*Sltrypsin1*/SPc and ds*Sltrypsin7*/SPc groups than in the dsGFP/SPc control group (Figure 6C). The pupation rates (containing malformed pupae) of *S. litura* treated with ds*Sltrypsin1*/SPc and ds*Sltrypsin7*/SPc decreased by 23.3% and 17.5%, respectively, compared to the dsGFP/SPc group (Figure 6D). The pupal malformation rate significantly increased by 60.2% and 57.0% following treatment with ds*Sltrypsin1*/SPc and ds*Sltrypsin7*/SPc, respectively (Figure 6E), compared to the control group, and normally developed pupae decreased by 40.5% and 42.4%, respectively. The pupal weight decreased significantly compared to the control group (Figure 6F). The eclosion rate treated with ds*Sltrypsin1*/SPc and ds*Sltrypsin7*/SPc decreased by 12.7% and 26.0%, respectively, compared to the control group (Figure 6G). The control efficacy of ds*Sltrypsin1*/SPc and ds*Sltrypsin7*/SPc was 48.1% and 57.4%, respectively.

**Figure 7:**
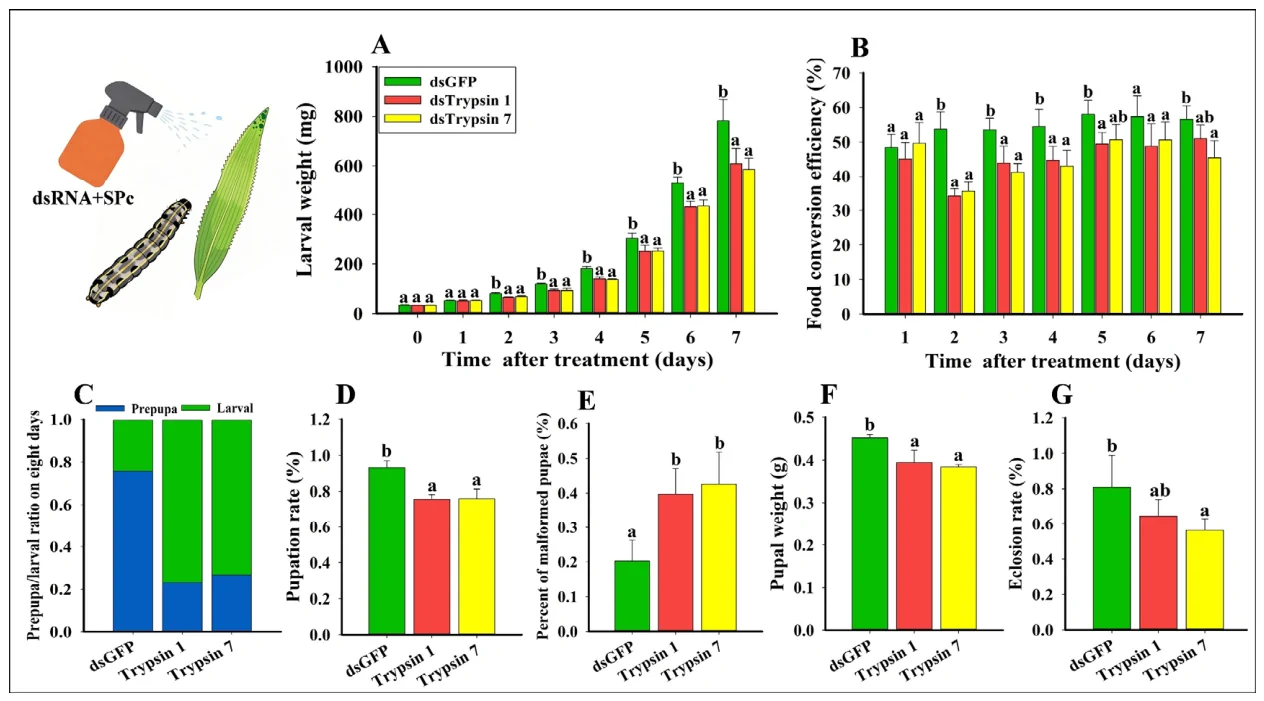
The influence on the development of *S. litura* after spraying dsSltrypsin1/SPc or dsSltrypsin7/SPc on maize leaves. (**A**) The monitored larval weight after spraying dsSltrypsin1/SPc or dsSltrypsin7/SPc for 7 days. (**B**) The food conversion efficiency of maize leaves after spraying dsSltrypsin1/SPc or dsSltrypsin7/SPc for 1, 2, 3, 4, 5, 6, and 7 days. (**C**) The prepupa/larval ratio of *S. litura* after spraying dsSltrypsin1/SPc or dsSltrypsin7/SPc for 8 days. The pupation rate of *S. litura* larvae (**D**), the percentage of malformed pupae (**E**), pupal weight (**F**) and eclosion rate (**G**) after spraying dsSltrypsin1/SPc or dsSltrypsin7/SPc. Different lowercase letters above the bars indicate significant differences analyzed by ANOVA followed by Tukey’s HSD test (*p* < 0.05).

Compared with larvae fed maize leaves sprayed with dsGFP/SPc, the weight of larvae treated with ds*Sltrypsin1*/SPc or ds*Sltrypsin7*/SPc decreased significantly compared to the 2nd day (Figure 7A). The food conversion efficiency of maize leaves treated with ds*Sltrypsin1*/SPc was significantly lower than that of dsGFP/SPc after 2, 3, 4, and 5 d, while that of ds*Sltrypsin7*/SPc was lower after 2, 3, 4, and 7 days (Figure 7B). The prepupal/larvae ratio in ds*Sltrypsin1*/SPc and ds*Sltrypsin7*/SPc treatments significantly decreased after 8 days compared to that in the dsGFP/SPc group (Figure 7C). The pupation rates (containing malformed pupae) of *S. litura* sprayed with ds*Sltrypsin1*/SPc and ds*Sltrypsin7*/SPc decreased by 18.9% and 18.6%, respectively, compared to the control group (Figure 7D). The malformed ratio caused by ds*Sltrypsin1*/SPc and ds*Sltrypsin7*/SPc significantly increased by 49.2% and 52.6%, respectively (Figure 7E) compared to the control group, and normally developed pupae decreased by 37.9% and 37.1%, respectively. The pupal weight decreased significantly compared to the control (Figure 7F). The eclosion rate treated with ds*Sltrypsin1*/SPc and ds*Sltrypsin7*/SPc decreased by 20.2% and 30.4%, respectively, compared to the control group (Figure 7G). The control efficacy of ds*Sltrypsin1*/SPc and ds*Sltrypsin7*/SPc was 50.5% and 56.2%, respectively.

### 3.7. Susceptibility of S. litura to Insecticides After Trypsin Gene Silencing

The synergistic effect of dsSltrypsin1/SPc and dsSltrypsin7/SPc on the toxicity of insecticides was determined by an artificial diet feeding assay. All LC_50_ values decreased to varying degrees (Table 1). The dsSltrypsin1/SPc treatment significantly increased the susceptibility of larvae to lufenuron, spinosad, and RH-5849, with synergist ratios (SR) of 1.957-, 1.571-, and 1.545- fold, respectively. The dsSltrypsin7/SPc treatment increased the susceptibility of larvae to chlorantraniliprole significantly, with an SR of 1.764- fold, while changes in susceptibility to other insecticides by dsSltrypsin1/SPc or dsSltrypsin7/SPc treatment were not significant.

## 4. Discussion

Trypsin plays a crucial role in insect growth, development and resistance to insecticides [26,27]. Silencing of trypsin3 in Chironomus kiiensis increased the susceptibility to abamectin significantly [28]. Trypsin inhibitor AeTI resulted in a decrease in both the larval body weight and survival of *S. litura* early instar larvae [29]. Given the important roles of trypsins in insects, we determined the influence on *S. litura* growth after silencing the expression of seven trypsin genes by RNAi and evaluated their potential in *S. litura* control.

The temporal-spatial expression characteristic of genes could reflect their function. For example, the molting-related trypsin homologs, *NlTrypsin-29* and *NlTrypsin-32* in *N. lugens,* were mainly expressed in the integument [10]. Here, the seven trypsin genes were highly expressed during the larval stage and mainly expressed in the larval gut, which indicated their possible role of digestion function. These results were consistent with the expression characteristics of trypsin genes in *Grapholita molesta* and *P. xylostella* [27,30,31]. The expression of insect trypsin genes is regulated by various factors, including hormones and developmental stages, resulting in complex regulatory mechanisms. In *H. armigera*, the expression of trypsin genes Ha-TLP and Ha-TLP2 was induced by the JH analog methoprene but suppressed by 20-hydroxyecdysone (20E) [32,33]. In *Schistocerca gregaria*, the trypsin-like serine protease Scg-SPRP exhibited significant expression fluctuations during molting and oviposition, being induced by JH III [34]. JH III treatment induced early trypsin expression in *Aedes aegypti* [35] and promoted the splicing of early trypsin pre-mRNA into mature mRNA, thereby regulating its transcriptional expression in *Culex quinquefasciatus* [36]. Here, the expression of most trypsin genes was induced by methoprene at low concentrations, but inhibited at high concentrations. The expression of trypsin genes was inhibited by geraniol. These results indicated that the expression of most selected trypsin genes might be positively correlated with JH titer, although further studies are required to determine whether this regulation is direct or indirect.

RNAi is a useful tool to study gene function and is being adopted to develop RNA biopesticides, which was recognized as one of the biggest scientific breakthroughs in 2024 [37]. But RNAi efficiency varies among different insect orders, which is generally high in Coleoptera but low in Lepidoptera [3,38], which limits their application in Lepidoptera. To solve this problem, multiple nanocarriers have been developed to efficiently deliver dsRNA in Lepidoptera [39,40,41,42]. SPc has been used to effectively silence genes in *Grapholita molesta* [43] and *Mythimna separata* [44], and to establish the RNAi system in all developmental stages of *S. frugiperda* [14]. In the *S. frugiperda* larval stage, SPc-loaded dsRNA could decrease gene expression by more than 50% in the 4th and 6th instar larvae by oral feeding with 2 μg and 3 μg dsRNA, respectively. The gene expression tended to revert to normal levels within 72 h, and this recovery of gene expression could be achieved by repeated RNAi operations every 24 h [14]. In this study, *S. litura* larvae fed with artificial diets containing 2 μg SPc-loaded dsRNA showed effective trypsin silencing at 24 h (33.4–54.6%), which had almost returned to control levels by 48 h. In addition to the generally low RNAi efficiency in lepidopteran pests, this may also be related to the fact that trypsin, as an important digestive enzyme, exhibits high abundance and constitutive expression, leading to rapid recovery of its expression levels shortly after dsRNA silencing. These results were consistent with the findings reported by Chao et al. [14]. Therefore, we replaced the artificial diets, or host leaves, every 24 h. Silencing of the seven trypsin genes in this study showed no influence on larval molting, but the successful silencing of *Sltrypsin1*, *Sltrypsin5* and *Sltrypsin7* significantly delayed pupation and inhibited larval/pupal weight, food conversion efficiency, and pupation rate, suggesting their important role in food digestion. Silencing of *Gmtrypsin9* in *G. molesta* also resulted in delayed pupation, decreased pupal weight and pupation rate [30]. Silencing multiple trypsin genes in *Bactrocera dorsalis* (Hendel) significantly reduced larval body length, weight, and overall size [26]. These results suggested that *Sltrypsin1* and *Sltrypsin7* played important roles in digestion. The field-simulating trial by spraying ds*Sltrypsin1*/SPc or ds*Sltrypsin7*/SPc also inhibited larval and pupal weight, which confirmed their important role in *S. litura* digestion of both soybean and maize. This trial also suggested that spraying ds*Sltrypsin1*/SPc or ds*Sltrypsin7*/SPc led to significant inhibition in food conversion efficiency (around 30%) and development parameters (pupation rate, malformation rate, eclosion rate). The comprehensive control efficacy was 48.1–57.4%, indicating that these two genes have good application potential in *S. litura* control. Although these experiments suggested that silencing of *Sltrypsin1* or *Sltrypsin7* showed a significant inhibitory effect on the growth and development of *S. litura*, they required continuous dsRNA treatment, making it unsuitable for field applications. In addition, dsRNA degradation, cellular uptake limitations, and saturation effect of the RNAi pathway resulted in low or unstable bioactivity of single-target RNA biopesticides [45,46]. It was observed that the interval between dsRNA treatment and pest death was at least a week [5,47]. To address these problems, an efficient and synergistic multicomponent nanopesticide containing pesticides, a nanocarrier and dsRNA was constructed. In *Lasioderma serricorne*, the silencing of *CYP4G249*, an essential gene for cuticular hydrocarbon biosynthesis, resulted in 29% larval mortality. Additionally, 24% of the larvae experienced difficulty during pupation and subsequently died. After silencing of *CYP4G249*, the mortality rate of ethyl formate and methyl isothiocyanate at LC_30_ increased by 33% and 29%, respectively [48]. In *Myzus persicae*, SPc-loaded *dsDpp* caused 45% mortality. Combined application of spirotetramat/SPc/dsDpp caused 94.5–96.6% mortality [4]. Here, we investigated the synergistic effects of combined application of insecticides with *Sltrypsin1*/SPc or *Sltrypsin7*/SPc, applied only once. Results demonstrated that silencing of *Sltrypsin1* or *Sltrypsin7* increased susceptibility to insecticides, especially to lufenuron, spinosad, RH-5849, and chlorantraniliprole. This might result from reduced digestive capacity caused by decreased trypsin activity, which in turn lowers the feeding desire and reduces food intake in insects, leading to nutritional deficiency and impaired growth and development, thereby increasing larval susceptibility. In addition, trypsins may also participate in other physiological functions, and their inhibition could cause specific physiological damage that renders larvae more sensitive to certain insecticides.

Trypsin was also reported to play important roles in Bt resistance. The Bt Cry proteins are formed as protoxins with no toxicity, and trypsin participates in their activation [49,50,51]. The reduced conversion of Bt protoxins to activated toxins by trypsin resulted in Bt resistance [50,52]. Previous studies also identified a series of trypsin proteins involved in Bt resistance in different lepidopteran insects, including OnT23 (*Ostrinia nubilalis*), SfT6 (*S. frugiperda*), PxTryp_SPc, HaSP2 (*H. armigera*), and HaTryR [27,53,54,55,56]. Here, silencing of *Sltrypsin1* or *Sltrypsin7* decreased the body weight of Bt-treated larvae significantly, indicating increased Bt toxicity (Figure S1). These results suggested that *Sltrypsin1* or *Sltrypsin7* did not participate in Bt activation and that their silencing did not reduce the insecticidal activity of the transgenic soybean and maize, indicating that they could be used compatibly with transgenic Bt crops.

## 5. Conclusions

This study identified the important roles of Sltrypsin1 and Sltrypsin7 in digestion. They could also be co-applied with insecticides to manage *S. litura* resistance. This study provides new insight into the application of trypsin genes in pest management.

## Figures and Tables

**Table 1:** The LC_50_ values of *S. litura* larvae treated with insecticides and dsSltrypsin1/SPc or dsSltrypsin7/SPc.

Insecticides	LC_50_ (95%FL, mg/kg)	Y = a + bX	Chi-Square	Df	R^2^	SR ^1^
Lufenuron	1.100 (0.807–1.490)	Y= −0.08 + 1.89X	3.421	3	0.941	-
dsSltrypsin1/SPc	0.562 (0.396–0.761)	Y= 0.47 + 1.89X	1.306	3	0.975	1.957
dsSltrypsin7/SPc	0.734(0.510–1.015)	Y= 0.23 + 1.69X	5.726	3	0.908	1.499
Indoxacarb	1.280 (1.010–1.640)	Y= −0.29 + 2.71X	2.581	3	0.968	-
dsSltrypsin1/SPc	0.875 (0.677–1.120)	Y= 0.15 + 2.56X	0.903	3	0.990	1.463
dsSltrypsin7/SPc	0.874 (0.684–1.110)	Y= 0.16 + 2.77X	0.733	3	0.991	1.464
Spinosad	28.191 (22.456–35.676)	Y= −2.39 + 1.71X	7.714	4	0.921	-
dsSltrypsin1/SPc	17.940 (15.144–21.107)	Y= −3.50 + 2.87X	4.817	4	0.957	1.571
dsSltrypsin7/SPc	20.229 (16.343–24.793)	Y= −3.23 + 2.45X	3.595	4	0.963	1.393
RH-5849	14.869 (12.355–17.782)	Y= −3.02 + 2.62X	5.357	4	0.955	-
dsSltrypsin1/SPc	9.623 (8.283–11.176)	Y= −4.19 + 4.25X	0.621	4	0.997	1.545
dsSltrypsin7/SPc	13.339 (11.296–15.699)	Y= −3.93 + 3.43X	5.687	4	0.963	1.115
Emamectin benzoate	0.055 (0.043–0.068)	Y= 3.64 + 2.84X	3.224	4	0.934	-
dsSltrypsin1/SPc	0.036 (0.023–0.048)	Y= 2.94 + 2.04X	0.828	4	0.978	1.528
dsSltrypsin7/SPc	0.040 (0.027–0.052)	Y= 2.90 + 2.07X	1.268	4	0.960	1.375
Chlorantraniliprole	0.194 (0.142–0.265)	Y= 0.97 + 1.34X	3.724	4	0.917	-
dsSltrypsin1/SPc	0.116 (0.080–0.154)	Y= 1.43 + 1.51X	6.099	4	0.889	1.672
dsSltrypsin7/SPc	0.110 (0.079–0.142)	Y= 1.67 + 1.73X	4.609	4	0.926	1.764
Chlorpyrifos	4.162 (3.563–4.875)	Y= −2.31 + 3.63X	1.687	4	0.999	-
dsSltrypsin1/SPc	3.530 (3.016–4.131)	Y= −2.08 + 3.73X	1.462	4	0.990	1.179
dsSltrypsin7/SPc	3.739 (3.243–4.309)	Y= −2.89 + 4.98X	0.354	4	0.997	1.113
Dinotefuran	13.206 (10.705–15.928)	Y= −3.26 + 2.89X	0.824	4	0.993	-
dsSltrypsin1/SPc	9.287 (7.628–10.973)	Y= −3.64 + 3.77X	0.275	4	0.997	1.422
dsSltrypsin7/SPc	9.384 (7.528–11.230)	Y= −3.07 + 3.15X	0.776	4	0.991	1.407

^1^ SR (synergist ratio) = LC_50_ value of insecticide/LC_50_ value of insecticide + dsSltrypsin.

## Data Availability

The original data are included in the paper. Further inquiries can be directed to the corresponding authors.

## Supplementary Materials

The following supporting information can be downloaded at https://www.mdpi.com/article/10.3390/insects17080861/s1. Figure S1: The larval weight of *S. litura* after being fed artificial diets containing Bt and dsSltrypsin1/SPc or dsSltrypsin7/SPc. Table S1: Primer pairs of Sltrypsin genes used for qRT-PCR; Table S2. Primer pairs for dsRNA synthesis of Sltrypsin genes.

## References

[1] Chen Y., Schutter K. (2024). Biosafety aspects of RNAi-based pests control. Pest Manag. Sci..

[2] Sergi E., Narduzzo A., Canito A.D., Favaretto F., Nerva L., Chitarra W., Bologna L., Bacenetti J., Maddalena G., Masiero S. (2026). RNA interference in crop protection: Opportunities and challenges during the transition to commercialization. Pest Manag. Sci..

[3] Fan C., Li Z., Xu Z., Yang Y., Qian X. (2026). RNAi-based molecular biopesticide: Inspired by nature, precision by science, and harmony to the ecosystem. J. Agric. Food Chem..

[4] Jiang Q., Dong M., Che L., Li C., Zhang X., Yin M., Shen J., Yan S. (2025). Standard strategy for developing multicomponent nanopesticides: From gene function analysis to co-delivery nano-platform construction. Chem. Eng. J..

[5] Cedden D., Bucher G. (2025). The quest for the best target genes for RNAi-mediated pest control. Insect Mol. Biol..

[6] Kong X., Tan S., Guan M., Lin X., Shen J., Shi W., Wang D. (2025). Nanocarrier-mediated transdermal delivery of Lmidgf4 dsRNA expedites biological control of locusts by Beauveria bassiana. J. Nanobiotechnol..

[7] Kuwar S.S., Pauchet Y., Vogel H., Heckel D.G. (2015). Adaptive regulation of digestive serine proteases in the larval midgut of Helicoverpa armigera in response to a plant protease inhibitor. Insect Biochem. Mol. Biol..

[8] Jelica L., Milena J.T. (2015). Dietary and phylogenetic correlates of digestive trypsin activity in insect pests. Entomol. Exp. Appl..

[9] Singh S., Singh A., Kumar S., Mittal P., Singh I.K. (2020). Protease inhibitors: Recent advancement in its usage as a potential biocontrol agent for insect pest management. Insect Sci..

[10] Xie Y., Zhang H., Li H., Zhang X., Luo X., Jiang M., Zhang C. (2023). Molting-related proteases in the brown planthopper, *Nilaparvata lugens*. Insect Biochem. Mol. Biol..

[11] Hou Y., Yang L., Xu S., Zhang Y., Cheng Y., Li Y., Gong J., Xia Q. (2021). Trypsin-type serine protease p37k hydrolyzes CPAP3-type cuticle proteins in the molting fluid of the silkworm Bombyx mori. Insect Biochem. Mol. Biol..

[12] Peng L., Zheng J., Liu L., Huang M., Cao M., Cui J., Vasseur L., You M., Zou M. (2025). Identification of seminal fluid proteins and reproductive function of trypsin-1 in male *Plutella xylostella*. Int. J. Biol. Macromol..

[13] Yan S., Yin M., Shen J. (2023). Nanoparticle-based nontransformative RNA insecticides for sustainable pest control: Mechanisms, current status and challenges. Entomol. Gen..

[14] Chao Z., Ma Z., Zhang Y., Yan S., Shen J. (2023). Establishment of star polycation-based RNA interference system in all developmental stages of fall armyworm *Spodoptera frugiperda*. Entomol. Gen..

[15] Li M., Ma Z., Peng M., Li L., Yin M., Yan S., Shen J. (2022). A gene and drug co-delivery application helps to solve the short life disadvantage of RNA drug. Nano Today.

[16] Ma Z., Zhang Y., Li M., Chao Z., Du X., Yan S., Shen J. (2022). A first greenhouse application of bacteria-expressed and nanocarrier-delivered RNA pesticide for *Myzus persicae* control. J. Pest Sci..

[17] Zhang Y., Ma Z., Zhou H., Chao Z., Yan S., Shen J. (2021). Nanocarrier-delivered dsRNA suppresses wing development of green peach aphids. Insect Sci..

[18] Dhir B.C., Mohapatra H.K., Senapati B. (1992). Assessment of crop loss in groundnut due to tobacco caterpillar, *Spodoptera litura* (F.). Indian J. Plant Prot..

[19] Saleem M.A., Ahmad M., Ahmad M., Aslam M., Sayyed A.H. (2008). Resistance to selected organochlorin, organophosphate, carbamate and pyrethroid, in *Spodoptera litura* (Lepidoptera: Noctuidae) from Pakistan. J. Econ. Entomol..

[20] Saleem M.A., Hussain D., Ghouse G., Abbas M., Fisher S.W. (2016). Monitoring of insecticide resistance in *Spodoptera litura* (Lepidoptera: Noctuidae) from four districts of Punjab, Pakistan to conventional and new chemistry insecticides. Crop Prot..

[21] Tong H., Su Q., Zhou X., Bai L. (2013). Field resistance of *Spodoptera litura* (Lepidoptera: Noctuidae) to organophosphates, pyrethroids, carbamates and four newer chemistry insecticides in Hunan, China. J. Pest Sci..

[22] Wang X., Huang Q., Hao Q., Ran S., Wu Y., Cui P., Yang J., Jiang C., Yang Q. (2018). Insecticide resistance and enhanced cytochrome P450 monooxygenase activity in field populations of *Spodoptera litura* from Sichuan, China. Crop Prot..

[23] Li D., He C., Wang M., Liu H., Liu R., Xu L. (2022). Toxicity of ribavirin to *Spodoptera litura* by inhibiting the juvenile hormone. J. Agric. Food Chem..

[24] Tang T. (2012). Silencing of Several Components of Cytochrome P450 Enzyme Systems in Helicoverpa armigera (Hübner) Resistant to Fenvalerate by RNA Interference.

[25] Fraenkel G. (1955). Inhibitory effects of sugars on the growth of the mealworm, *Tenebrio molitor* L.. J. Cell. Comp. Physiol..

[26] Li Y., Hou M., Shen G., Lu X., Wang Z., Jia F., Wang J., Dou W. (2017). Functional analysis of five trypsin-like protease genes in the oriental fruit fly, Bactrocera dorsalis (Diptera: Tephritidae). Pestic. Biochem. Physiol..

[27] Gong L., Kang S., Zhou J., Sun D., Guo L., Qin J., Zhu L., Bai Y., Ye F., Akami M. (2020). Reduced expression of a novel midgut trypsin gene involved in protoxin activation correlates with Cry1Ac resistance in a laboratory-selected strain of *Plutella xylostella* (L.). Toxins.

[28] Zheng X., Li Q., Ullah F., Lu Z., Mo W., Guo J., Liu X., Xu H., Lu Y. (2024). Abamectin exposure causes chronic toxicity and trypsin/chymotrypsin damages in *Chironomus kiiensis* Tokunaga (Diptera: Chironomidae). Pestic. Biochem. Physiol..

[29] Bhattacharyya A., Leighton S.M., Babu C.R. (2007). Bioinsecticidal activity of Archidendron ellipticum trypsin inhibitor on growth and serine digestive enzymes during larval development of *Spodoptera litura*. Comp. Biochem. Physiol. Part C Toxicol. Pharmacol..

[30] Lv D., Kassen K., Men C., Yang X., Pan D., Wang X., Wang N., Wang P., Yuan X., Li Y. (2024). Trypsin-encoding gene function of efficient star polycation nanomaterial-mediated dsRNA feeding delivery system of *Grapholita molesta*. Pest Manag. Sci..

[31] Lü D., Pan D., Wang D., Ye J., Sha X., Zhang X., Fu Y., Yuan X., Li Y. (2025). Study on the functional role and tissue localization of GmolTrypsin in larvae of Grapholita molesta feeding on different host plants. Insect Biochem. Mol. Biol..

[32] Liu Y., Sui Y., Wang J., Zhao X. (2009). Characterization of the Trypsin-like protease (Ha-TLP2) constitutively expressed in the integument of the cotton bollworm, *Helicoverpa armigera*. Arch. Insect Biochem. Physiol..

[33] Sui Y., Wang J., Zhao X. (2009). The impacts of classical insect hormones on the expression profiles of a new digestive trypsin-like protease (TLP) from the cotton bollworm, *Helicoverpa armigera*. Insect Mol. Biol..

[34] Chiou S., Broeck J., Janssen I., Borovsky D., Vandenbussche F., Simonet G., De Loof A. (1998). Cloning of the cDNA encoding Scg-SPRP, an unusual ser-protease-related protein from vitellogenic female desert locusts (*Schistocerca gregaria*). Insect Biochem. Mol. Biol..

[35] Zhu J., Busche J., Zhang X. (2010). Identification of juvenile hormone target genes in the adult female mosquitoes. Insect Biochem. Mol. Biol..

[36] Borovsky D., Hancock R., Rougé P., Powell C., Shatters R.G. (2018). Juvenile hormone affects the splicing of *Culex quinquefasciatus* early trypsin messenger RNA. Arch. Insect Biochem. Physiol..

[37] Stokstad E. (2024). RNA-based pesticides enter the field. Science.

[38] Ullah F., Guru Pirasanna Pandi G., Yan S., Haider K., Sarangi S., Gul H., Li X., Guedes R.N.C., Desneux N., Lu Y. (2026). Nano-enabled RNA interference: Emerging trends in the sustainable management of lepidopteran pests. J. Pest Sci..

[39] Christiaens O., Tardajos M.G., Reyna Z.L.M., Dash M., Dubruel P., Smagghe G. (2018). Increased RNAi Efficacy in *Spodoptera exigua* via the formulation of dsRNA with guanylated polymers. Front. Physiol..

[40] Martinez Z., De Schutter K., Van Damme E.J., Vogel E., Wynant N., Vanden Broeck J., Christiaens O., Smagghe G. (2021). Accelerated delivery of dsRNA in lepidopteran midgut cells by a Galanthus nivalis lectin (GNA)-dsRNA-binding domain fusion protein. Pestic. Biochem. Physiol..

[41] Gurusamy D., Mogilicherla K., Shukla J.N., Palli S.R. (2020). Lipids help double-stranded RNA in endosomal escape and improve RNA interference in the fall armyworm, *Spodoptera frugiperda*. Arch. Insect Biochem. Physiol..

[42] Ma Z., Zheng Y., Chao Z., Chen H., Zhang Y., Yin M., Shen J., Yan S. (2022). Visualization of the process of a nanocarrier-mediated gene delivery: Stabilization, endocytosis and endosomal escape of genes for intracellular spreading. J. Nanobiotechnol..

[43] Wei H., Tan S., Yan S., Li Z., Shen J., Liu X. (2022). Nanocarrier-mediated transdermal dsRNA-NPF1 delivery system contributes to pest control via inhibiting feeding behavior in Grapholita molesta. J. Pest Sci..

[44] Li W., Liao J., Li L., Dai C., Hu Y., Qian Y., Li H. (2026). Upregulation of two cuticular proteins is associated with resistance to *Beauveria bassiana* in crowded *Mythimna separata*. Insects.

[45] Dietz-Pfeilstetter A., Mendelsohn M., Gathmann A., Klinkenbuss D. (2021). Considerations and regulatory approaches in the USA and in the EU for dsRNA-based externally applied pesticides for plant protection. Front. Plant Sci..

[46] Lu J., Shen J. (2024). Target genes for RNAi in pest control: A comprehensive overview. Entomol. Gen..

[47] Cedden D., Güney G., Scholten S., Rostás M. (2024). Lethal and sublethal effects of orally delivered double-stranded RNA on the cabbage stem flea beetle, *Psylliodes chrysocephala*. Pest Manag. Sci..

[48] Liang C., Qin D., Yang W., Li C., Wan F., Smagghe G., Xu K. (2026). MicroRNA *Lse-novel_mir63* regulates metamorphosis and fumigant tolerance by targeting *CYP4G249* in *Lasioderma serricorne*. Pest Manag. Sci..

[49] Adang M.J., Crickmore N., Jurat-Fuentes J.L. (2014). Diversity of *Bacillus thuringiensis* crystal toxins and mechanism of action. Adv. Insect Physiol..

[50] Oppert B., Kramer K.J., Beeman R.W., Johnson D., McGaughey W.H. (1997). Proteinase-mediated insect resistance to *Bacillus thuringiensis* toxins. J. Biol. Chem..

[51] Lightwood D.J., Ellar D.J., Jarrett P. (2000). Role of proteolysis in determining potency of *Bacillus thuringiensis* Cry1Ac δ-endotoxin. Appl. Environ. Microbiol..

[52] Wu Y. (2014). Detection and mechanisms of resistance evolved in insects to Cry toxins from *Bacillus thuringiensis*. Adv. Insect Physiol..

[53] Liu C., Xiao Y., Li X., Oppert B., Tabashnik B.E., Wu K. (2014). Cis-mediated down-regulation of a trypsin gene associated with Bt resistance in cotton bollworm. Sci. Rep..

[54] Li H., Oppert B., Higgins R.A., Huang F., Buschman L.L., Gao J.-R., Zhu K.Y. (2005). Characterization of cDNAs encoding three trypsin-like proteinases and mRNA quantitative analysis in Bt-resistant and -susceptible strains of *Ostrinia nubilalis*. Insect Biochem. Mol. Biol..

[55] Rodríguez-Cabrera L., Trujillo-Bacallao D., Borrás-Hidalgo O., Wright D.J., Ayra-Pardo C. (2010). RNAi-mediated knockdown of a *Spodoptera frugiperda* trypsin-like serine-protease gene reduces susceptibility to a *Bacillus thuringiensis* Cry1Ca1 protoxin. Environ. Microbiol..

[56] Rajagopal R., Arora N., Sivakumar S., Rao N.G.V., Nimbalkar S.A., Bhatnagar R.K. (2009). Resistance of *Helicoverpa armigera* to Cry1Ac toxin from *Bacillus thuringiensis* is due to improper processing of the protoxin. Biochem. J..

