# Supplementary material for: Functional Characterization of Trypsin Genes in Spodoptera litura and Their RNA Silencing in Combination with Insecticides
Source: Insects. 2026 Aug 18;17(8):861. doi: 10.3390/insects17080861 (PMC13513355; doi:10.3390/insects17080861)
Supplement: Supplementary file 1 [file insects-17-00861-s001.zip › insects-4459129-supplementary.pdf]

Functional Characterization of Trypsin Genes in *Spodoptera litura* and Their RNA Silencing in Combination with Insecticides

Li Xu[1,2,3], Ke Wang[1,2,3], Yin Huang[1,2,3], Yunsheng Wei[1,2,3], Hao Yu[1,2,3], Runqiang Liu[1,2,3*], Dongzhi Li[1,2,3*]

[1] College of Plant Protection and Environment, Henan Institute of Science and Technology, Xinxiang, 453003, Henan Province, China.

[2] Henan Engineering Research Center of Green Pesticide Creation & Intelligent Pesticide Residue Sensor Detection, Henan Institute of Science and Technology, Xinxiang, 453003, Henan Province, China.

[3] State Key Laboratory of High-Efficiency Production of Wheat-Maize Double Cropping, Xinxiang, 453003, Henan Province, China.

*Corresponding authors:

Runqiang Liu, E-mail: liurunqiang1983@126.com;

Dongzhi Li, E-mail: lidongzhi1989@163.com.

Table S1. Primer pairs of Sltrypsin genes used for qRT-PCR

Gene ID	Gene Name	Forward	Reverse
LOC111353055	Sltrypsin1	TGAGTTTATTCAGCCCGC	ACCAACAACGACACCATT
LOC111353056	Sltrypsin 2	GTCGTTCACAACGTCAACCAG	CCAGAGAAAGTGTCACCCCAT
LOC111353058	Sltrypsin 3	CGCTCTTCTGCGTGTTAGTG	CGGAATGCGTATCTGTTTTT
LOC111353073	Sltrypsin 4	AGGTTCCTCCTTCGCTAA	CAGACGGGCTGATTTTCG
LOC111353079	Sltrypsin 5	ACAGAGGATTGTAGGTGG	CAGAAGTAAGGATGAACG
LOC111356395	Sltrypsin 6	GCTGTCGTCGCTTTACCT	CGCTTGCACAAACATTTG
LOC111348269	Sltrypsin 7	CACCGTGGACCAATACCC	ACCGCTGGACGCAAAAGA
KC007373.1	EF1α	CGAAACCGCTAAATACTATGTCAC	AACGGGATTCGCTGTATGG
KC866373	RPL10	GACTTGGGTAAGAAGAAG	GATGACATGGAATGGATG

Table S2. Primer pairs for dsRNA synthesis of Sltrypsin genes

Gene name	Primers
Sltrypsin1-F	GGGCCCTGCCGGTGCCATTCTAAA
Sltrypsin1-R	GAGCTCCACGTGACGCAGCTGTGC
Sltrypsin2-F	GGGCCCGACCGATACCCCACCATT
Sltrypsin2-R	GAGCTCGTGTCACCCCATCCAGCA
Sltrypsin3-F	GGGCCCAGCGGTCCCCTACAATCC
Sltrypsin3-R	GAGCTCCCCATCCTGCTACCCAGA
Sltrypsin4-F	GGGCCCACCATTAACCAGTACCCGCA
Sltrypsin4-R	GAGCTCCCAGAGAAAGTATCACCCCA
Sltrypsin5-F	GGGCCCAGAGGATTGTAGGTGGCA
Sltrypsin5-R	GAGCTCGGGAGAATATAGTCGGGG
Sltrypsin6-F	GGGCCCCAGTTTCAAGCACTGCAAGACA
Sltrypsin6-R	GAGCTCACGATATAAGGTAAAGCGACGA
Sltrypsin7-F	GGGCCCTGTGGGGCATCTGGTGGTTC
Sltrypsin7-R	GAGCTCGGGTCTTCAGGTAGGCGTAG
GFP-F	GGGCCCGCAGTGCTTCAGCCGCTACCCC
GFP-R	GAGCTCATGTGATCGCGCTTCTCGTTGG

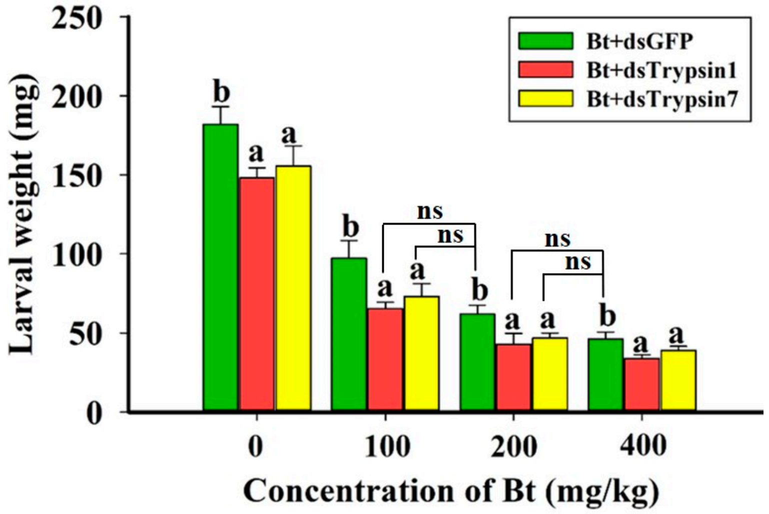

Figure S1. The larvae weight of *S. litura* after being fed with artificial diets containing Bt and dsSltrypsin1/SPc or dsSltrypsin7/SPc. Different lowercase letters above the bars indicated significant difference analyzed by ANOVA followed by Tukey's HSD test ($P < 0.05$). ns, not significant.
